# Regulatory T cells in renal disease

**DOI:** 10.1002/cti2.1004

**Published:** 2018-01-30

**Authors:** Maliha A Alikhan, Megan Huynh, A Richard Kitching, Joshua D Ooi

**Affiliations:** ^1^ Centre for Inflammatory Diseases Department of Medicine Monash University Monash Medical Centre Clayton Victoria Australia; ^2^ Department of Nephrology Monash Health Clayton VIC Australia; ^3^ Department of Paediatric Nephrology Monash Health Clayton VIC Australia

**Keywords:** acute kidney injury, autoimmune renal disease, chronic kidney disease, glomerulonephritis, intrarenal regulatory T cells, transplantation

## Abstract

The kidney is vulnerable to injury, both acute and chronic from a variety of immune and metabolic insults, all of which at least to some degree involve inflammation. Regulatory T cells modulate systemic autoimmune and allogenic responses in glomerulonephritis and transplantation. Intrarenal regulatory T cells (Tregs), including those recruited to the kidney, have suppressive effects on both adaptive and innate immune cells, and probably also intrinsic kidney cells. Evidence from autoimmune glomerulonephritis implicates antigen‐specific Tregs in HLA‐mediated dominant protection, while in several human renal diseases Tregs are abnormal in number or phenotype. Experimentally, Tregs can protect the kidney from injury in a variety of renal diseases. Mechanisms of Treg recruitment to the kidney include via the chemokine receptors CCR6 and CXCR3 and potentially, at least in innate injury TLR9. The effects of Tregs may be context dependent, with evidence for roles for immunoregulatory roles both for endogenous Tbet‐expressing Tregs and STAT‐3‐expressing Tregs in experimental glomerulonephritis. Most experimental work and some of the ongoing human trials in renal transplantation have focussed on unfractionated thymically derived Tregs (tTregs). However, induced Tregs (iTregs), type 1 regulatory T (Tr1) cells and in particular antigen‐specific Tregs also have therapeutic potential not only in renal transplantation, but also in other kidney diseases.

## Introduction

**Figure 1:**
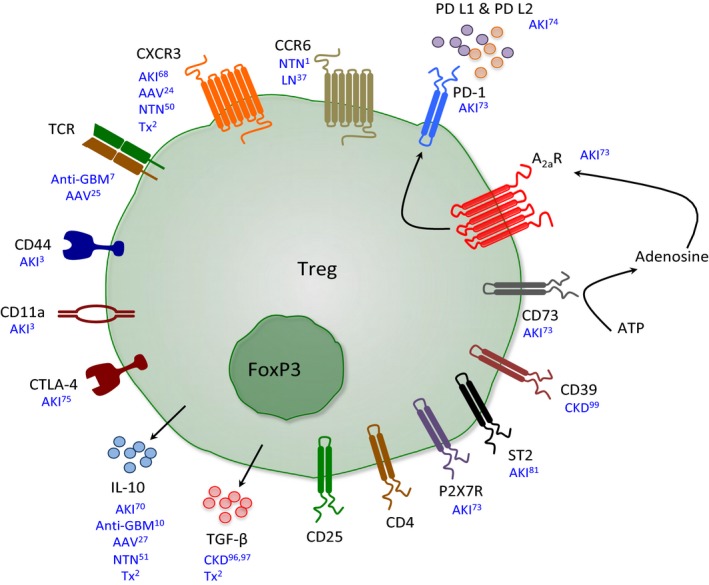
Important mechanisms used by Tregs to suppress inflammation in renal disease. A schematic diagram depicting some of the surface molecules that Tregs express and the cytokines they release to suppress inflammation that have been implicated in selected *in vivo* experimental models of different renal diseases. Renal Tregs constitutively express the transcription factor Foxp3 and surface molecules CD4 and CD25. AAV, ANCA‐associated vasculitides; AKI, acute kidney injury; Anti‐GBM, antiglomerular basement membrane disease; CKD, chronic kidney disease; LN, lupus nephritis; NTN, nephrotoxic serum nephritis; Tx, transplantation.

The kidney is a highly vascular organ important for maintaining internal homeostasis, including the removal of toxins from the blood. Its anatomical structure and function render it vulnerable to both immune‐ and nonimmune‐mediated injury. Although lymphocytes are uncommon in healthy kidneys, their numbers increase considerably in disease. This review will cover research into the role of Tregs in renal injury. The relevance of Tregs has been shown in several instances (see Figure 1). Firstly, as in other autoimmune diseases, Tregs are important in the maintenance of tolerance to autoantigens that are responsible for autoimmune renal disease (‘nephritogenic’ autoantigens). Secondly, Tregs play local roles within the kidney in dampening renal inflammation, both in responses that are clearly mediated by immune responses to nephritogenic antigens, and in inflammatory responses that are largely or solely ‘innate’, with little evidence for antigen‐specific responses. Thirdly, regulatory T cells are critical in preventing allogeneic responses, and in renal transplantation may be a key to transplant tolerance. The overall phenotype of intrarenal regulatory T cells remains poorly characterised, although evidence suggests a role for CCR6 and CXCR3,^1^, ^2^ and of the adhesion molecules CD11a and CD44 in their recruitment to the kidney in inflammatory diseases.^3^

## Regulatory T cells in autoimmune glomerulonephritis

An increasing number of forms of glomerulonephritis are now known to be autoimmune in origin, including anti‐glomerular basement membrane (anti‐GBM) glomerulonephritis, anti‐neutrophil cytoplasmic antibody‐associated vasculitis, lupus nephritis, ‘primary’ membranous nephropathy and IgA nephropathy.^4^ While autoantibodies mediate injury in many forms of autoimmune glomerulonephritis, effector CD4^+^ and CD8^+^ T cells also play a role, meaning that Tregs have multiple potential sites of action, from the maintenance or re‐establishment of tolerance systemically to the relevant nephritogenic autoantigens, to the suppression of adaptive and innate effectors of injury within the kidney. See Table 1 for a summary of the role of Tregs in renal injury.

**Table 1:** Models of renal disease where Tregs have been shown to be protective

Underlying immune response	Disease being modelled	Endogenous or transferred Tregs	References
Autoimmune nephritis	Goodpasture's disease	Endogenous	9
	MPO‐ANCA‐associated glomerulonephritis	Endogenous	25
	Lupus nephritis	Endogenous	37
	IgA nephropathy	Transferred	60
Foreign antigen	Masugi nephritis	Both	49, 55
Innate immunity/chronic kidney disease	Renal IRI	Both	68, 69, 70, 71, 72
	Cisplatin nephrotoxicity	Both	3, 84
	Adriamycin nephropathy	Both	94, 95
	Diabetic nephropathy	Both	106
Renal transplantation		Both	2, 118

### Autoimmune anti‐glomerular basement membrane disease (Goodpasture's disease)

Anti‐GBM disease results from autoimmunity against the noncollagenous domain of the α3‐chain of type IV collagen (α3[IV]NC1), a structural component of specialised basement membranes in the kidney and the lung. Rapidly progressive glomerulonephritis is a key characteristic of this disease but life‐threatening pulmonary haemorrhage also occurs.^5^ As in many autoimmune diseases, there are strong HLA associations, with further steps involved before T‐ and B‐cell tolerance is lost. Local systemic inflammatory events may unmask hidden or ‘cryptic’ B‐cell epitopes of the autoantigen, promoting loss of B cell tolerance, and allow access to pathogenic autoantibodies, that, together with cell‐mediated effectors, promote intense local inflammatory responses leading to severe glomerular injury.^6^

Tregs are major mediators of tolerance and the mechanism behind the HLA‐mediated dominant protection from the risk of anti‐GBM disease. The relative risk of developing this disease is markedly higher in those that bear the HLA‐DRB1*1501 (DR15) allele.^7^ This susceptibility has been modelled in HLA transgenic mice, in which anti‐GBM glomerulonephritis can be induced in humanised DR15 transgenic (Tg) mice by immunisation with the immunodominant CD4^+^ T‐cell epitope.^8^ Dominant protection is afforded by HLA‐DRB1*01 (DR1) and HLA‐DRB1*07 (DR7), in that epidemiologically, susceptibility is abrogated when either of these allomorphs are co‐expressed with DR15.^7^ Mechanistically, the DR1‐mediated protection *in vitro* in human and mouse systems, and *in vivo* in the HLA transgenic mouse system, is associated with and mediated by the presence of thymically derived Tregs (tTregs) specific for the immunodominant epitope.^9^ Further investigation into how HLA‐DR1 confers protection revealed, through crystal structures and single‐cell TCR sequencing of tetramer‐specific T cells, that DR1 presented the immunodominant Goodpasture's T‐cell autoepitope in a conformation that preferentially interacts with Tregs.^9^

Unusually, anti‐GBM disease does not follow the relapsing‐remitting disease course that characterises many autoimmune diseases. Despite the loss of tolerance to α3(IV)NC1 with often catastrophic consequences, relapse is rare, with Tregs potentially mediating restoration of tolerance to α3(IV)NC1, and preventing disease recurrence. Analysis of T cells from patients during acute and convalescent disease revealed a Treg population present during the later disease stage, suggesting Treg involvement in suppressing autoimmunity and the re‐establishment of tolerance to α3(IV)NC1,^10^ findings corroborated by additional patient data demonstrating reduced proliferative responses and increased IL‐10 production in late disease, independent of immunosuppressive treatment.^11^

### Anti‐neutrophil cytoplasmic antibody‐associated vasculitis

Anti‐neutrophil cytoplasmic antibody‐associated vasculitis (AAV) can be classified into different syndromic presentations, namely microscopic polyangiitis (MPA), granulomatosis with polyangiitis (GPA) and eosinophilic granulomatosis with polyangiitis, with MPA and GPA together being the most common cause of rapidly progressive glomerulonephritis.^12^ AAV is caused by the loss of tolerance to the neutrophil cytoplasmic antigens: myeloperoxidase (MPO), proteinase‐3 (PR3), and there is also evidence for loss of tolerance to lysosomal‐associated membrane protein 2 (LAMP‐2).^13^, ^14^, ^15^ Autoantibodies specific for PR3 or MPO bind to primed neutrophils leading to neutrophil activation.^16^ This results in neutrophils adhering to and migrating within the glomerular microvasculature where they induce glomerular endothelial injury damage by the release of reactive oxygen species and proteases. In this context, they also deposit the target autoantigens MPO and PR3, which then results in recruitment of effector CD4^+^ T cells and CD8^+^ cells that exacerbate and perpetuate disease.^17^, ^18^, ^19^

Human studies implicate abnormal Treg number and function in AAV. In GPA patients with active disease, those who achieved remission by 14 weeks presented with higher proportions of CD4^+^ Foxp3^+^ Tregs compared with patients who were slower to remit.^20^ Tregs from GPA patients in remission, although increased in proportion compared to healthy controls, had a decreased ability to suppress responder T cells,^20^, ^21^ while Tregs from AAV patients with active disease have an even poorer suppressive capacity than those from patients in remission.^22^ This decrease in suppressive function of Tregs from AAV patients could be explained by the finding that Tregs from AAV patients preferentially express a splice variant of Foxp3 lacking exon 2.^23^ Lastly, in kidney biopsies from AAV patients, CD4^+ ^Foxp3^+^ Tregs expressing the chemokine receptor CXCR3 were recruited to the kidney and found in direct cell–cell contact with CXCR3^+ ^Foxp3^−^ Th1 cells.^24^

Myeloperoxidase, a key autoantigen in AAV, is highly AIRE‐regulated, and AIRE‐deficient mice immunised with MPO develop enhanced autoimmunity to MPO. However, MPO‐AAV is not a feature of autoimmune polyendocrinopathy–candidiasis–ectodermal dystrophy/dysplasia (APECED). In experimental anti‐MPO glomerulonephritis, anti‐CD25 mAb Treg depletion enhanced anti‐MPO‐specific autoimmunity and exacerbated disease.^25^ Mechanisms that enhance Treg number and function, including IL‐10‐secreting mast cells,^26^ and nasal insufflation of an immunodominant CD4^+^ T‐cell MPO epitope, MPO_409‐428_, protect mice from experimental anti‐MPO autoimmunity and glomerulonephritis.^27^ Therefore, observational and experimental data in AAV suggest that strategies that enhance Treg function may lead to more targeted therapies.

### Lupus nephritis

Lupus nephritis frequently occurs in patients with systemic lupus erythematosus (SLE) and is a key determinant of outcome. Autoantibodies of multiple specificities can be deposited in the kidney, either as circulating or as *in situ* immune complexes. Effector T cells also play a role in inducing glomerular injury.^28^ Target autoantigens include nuclear antigens, which can be deposited in the kidney by neutrophils that undergo NETosis.^29^ The variable autoantigens and effector responses result in a range of patterns of glomerular injury. Studies of peripheral blood Tregs in patients with SLE have shown decreases in Treg numbers and defective Treg phenotypes.^30^ In patients with active lupus nephritis, urinary *FOXP3* mRNA is increased compared with patients with inactive lupus and healthy controls.^31^ This could be explained by a transient activation of Foxp3 in activated effector T cells in humans^32^, ^33^ or may conceptually be consistent with a regulatory cell:effector cell ratio (rather than an absolute number) being more relevant in tissue injury. In lupus nephritis, pilot data from paraffin embedded kidneys stained for Foxp3^+^ and CD3^+^ cells have shown that kidney sections from patients with lupus nephritis class IV (the most active and severe form) have lower proportions of Foxp3^+^/CD3^+^ cells compared to patients with class V (membranous) lupus nephritis.^34^

In murine lupus nephritis, the BWF1 and SNF1 strains have fewer CD4^+ ^CD25^+^ Tregs compared to normal BALB/c and DBA/1 mice.^35^ Although CD4^+ ^CD25^+^ Tregs in BWF1 mice could suppress CD4^+ ^CD25^−^ T‐cell proliferation, they did not suppress T cell‐mediated IgG production.^36^ Depletion studies in pristane‐induced models of lupus nephritis have demonstrated that endogenous Stat3‐expressing Foxp3^+^ Tregs (‘Treg17’) afford protection from injury, as Foxp3‐Cre deletion of Stat3 led to heightened Th17 responses and less Treg17 cell recruitment to the kidney, likely due to impaired CCR6 expression.^37^ The same group showed that in the same model, Foxp3^+^ RORγt^+^ Tregs (‘biTregs’) were pathogenic, at least in part, because RORγt induce the secretion of IL‐17 in biTreg cells.^38^

A number of treatments in several murine models of lupus have been associated with increased Treg number or function, including, in MRL/lpr strain, IL‐33 inhibition, piperlongumine and the 4‐hydroxyquinoline‐3‐formamide derivative (known as Y27)^39^, ^40^, ^41^; in NZBxW/F1 mice, IL‐2/IL‐2 mAb immune complexes, G‐CSF and tuftsin‐phosphorylcholine^42^, ^43^, ^44^; and in the bm12→B6 chronic graft‐versus‐host model of lupus nephritis, microRNA‐21 deficiency.^45^ Progesterone may also be important for optimal Treg number and function, as progesterone‐deficient Nba2 mice have increased antichromatin IgG and proteinuria associated with a decrease in Tregs.^31^

### Experimental rapidly progressive glomerulonephritis induced by foreign globulins

Masugi nephritis, also known as nephrotoxic serum nephritis or ‘anti‐GBM’ glomerulonephritis, is an experimental model of glomerulonephritis that is not autoimmune, but is induced by the deposition of heterologous antibodies on the GBM.^46^, ^47^, ^48^ Renal injury is mediated both by innate and adaptive immune responses against the deposited foreign antibody (as an antibody and as an antigen). While not a model of autoimmune disease, it has been used widely to understand immune‐mediated forms of glomerulonephritis and is particularly useful in studying effector responses. In this disease model, endogenous Tregs infiltrate the kidney over time, and depletion of these endogenous Tregs, in Foxp3^DTR^ (DEREG) mice, exacerbates glomerulonephritis, even during established disease.^49^, ^50^ Intrarenal flow cytometry revealed that some endogenous renal Tregs in nephritic mice produced IL‐10, with targeted deletion of Treg IL‐10 resulting in a modest exacerbation of renal injury.^51^ There is also evidence that intrarenal CD103^+^ dendritic cells (<5% of renal DCs) support the development and retention of Tregs.^52^ Th1‐driven autoimmunity is a key feature of this disease model, and CXCR3^+^ and Tbet^+^ Tregs have been shown to infiltrate the kidney and dampen specifically Th1‐driven glomerulonephritis.^24^, ^53^ These data are consistent with more recent studies that demonstrate an essential role for Tbet^+^ Tregs to suppress Th1‐driven autoimmunity.^54^ In this model of glomerulonephritis, transferring CD4^+ ^CD25^+^ Tregs prior to the injection of heterologous anti‐GBM antibodies attenuated glomerulonephritis,^55^ in part via secretion of IL‐9, which attracts immunosuppressive mast cells into lymph nodes.^56^ While the transfer of these CD4^+ ^CD25^+^ thymically derived Tregs protect, the transfer of *ex vivo* induced Tregs (iTregs), produced via several different protocols do not protect mice from renal disease. Significant proportions of these iTregs lose their expression of Foxp3 *in vivo*, likely acquiring effector function.^57^

### IgA Nephropathy

IgA nephropathy is the most common form of primary glomerulonephritis worldwide and is characterised by IgA deposition in the glomerular mesangium. Glomerular IgA immune complex deposition triggers innate immune responses and subsequent T‐cell activation and inflammation. In patients with IgA nephropathy, there may be an imbalance of Tregs and Th17 cells in the periphery and in renal tissues, with a lower frequency of CD45RA^− ^Foxp3^hi^‐activated Tregs and an increase in Th17 cells.^58^, ^59^ These differences are associated with reduced levels of serum IL‐10 and increased levels of serum and urine IL‐17A in IgA patients.^58^, ^59^ Furthermore, the altered distribution of Tregs and Th17 cells correlates with prognostic indicators such as impaired GFR, proteinuria, tubulointerstitial injury and hypertension.^58^ Functionally, a small study in rats with IgA nephropathy suggests that the adoptive transfer of CD4^+ ^CD25^+^ Tregs, expanded *in vitro*, reduces proteinuria and possibly IgA deposition, hyperplasia of glomerular mesangial cells and tubular epithelial damage.^60^

### Other forms of glomerulonephritis

Membranous glomerulopathy is the commonest cause of nephrotic syndrome in adults. The majority of cases of what was previously described as ‘idiopathic’ membranous nephropathy is now known to be due to autoimmunity to the phospholipase A2 receptor.^61^ Although there is a paucity of data in humans with this disease, numbers of Tregs in the peripheral blood have been reported to be decreased,^62^, ^63^ and early responsiveness to rituximab has been linked to an increase in the proportion of Tregs.^63^ In children, minimal change disease is the commonest cause of nephrotic syndrome. Although the pathogenesis of this disease is unclear, some lines of evidence imply a role for T lymphocytes, with some evidence for a role for Tregs from human descriptive studies and in animal models, reviewed by Bertelli *et al*.^64^

## Acute kidney injury

Acute kidney injury (AKI) is defined as an abrupt decline in renal function. It frequently occurs in hospitalised patients and the critically ill, and often in those with pre‐existing renal disease.^65^ While recovery from AKI is common, AKI greatly enhances the risk of developing chronic kidney disease and end‐stage renal disease.^66^ AKI has multiple aetiologies, with hypovolaemia, ischaemia–reperfusion injury (IRI), exposure to nephrotoxic agents and sepsis amongst the major causes. AKI includes a complex series of events leading to tubular injury, altered intrarenal haemodynamics and the activation of the immune system contributing to renal inflammation and dysfunction.^67^ While there are as yet little clinical data about Treg numbers and function in patients with AKI, there is compelling evidence for a potent protective role of Tregs in experimental AKI, suggesting that regulatory T cells may have therapeutic potential in the prevention or treatment of human AKI.

### Ischaemia–reperfusion injury

Renal IRI is a common complication of major surgery, for example cardiopulmonary bypass surgery. It is also an obligatory component of kidney transplantation that if severe, it leads both to delayed allograft function and an increase risk of acute transplant rejection. In naïve mice and 24 h after renal IRI, fewer than 1% of the resident leucocyte (CD45^+^) population in the kidney are CD4^+ ^Foxp3^+^ Tregs.^68^ However, during the repair phase of IRI, beginning 72 h after reperfusion, Tregs infiltrate the kidney and remain as long as 10 days after reperfusion.^69^ A large proportion of these Tregs (40%) express CXCR3, which is possibly involved in the trafficking of Tregs to the kidney following renal ischaemia.^68^ Treg depletion using anti‐CD25 monoclonal antibodies prior or within 24 h of IRI enhances renal inflammation, acute tubular necrosis and renal dysfunction, suggesting that intrinsic Tregs traffic to the kidney to promote repair after ischaemic injury.^68^, ^69^, ^70^, ^71^, ^72^ Furthermore, after IRI, kidneys from *Rag1* ^−/−^ mice reconstituted with Foxp3‐deficient lymph node cells exhibited more injury and more intrarenal leucocytes compared to mice reconstituted with wild‐type cells.^70^ Freshly isolated Tregs transferred 24 h after reperfusion traffic to the postischaemic kidney with accelerated recovery of tubular injury and renal function, as well as reduced CD4^+^ T‐cell TNF and IFN‐γ production.^69^ Treg depletion studies suggest that Tregs appear to modulate the inflammatory milieu via different actions depending on the stage of IRI, with Tregs limiting innate immune responses during the early phase of IRI and modulating CD4^+^ T‐cell responses (albeit with any antigen‐specific component being unclear) during the repair phase.^69^, ^70^

Tregs appear to suppress renal IRI through multiple mechanisms. Wild‐type, but not IL‐10‐deficient Treg transfer into *Rag1* ^*−/−*^ mice could limit renal IRI implicating IL‐10 production, in part, as a mechanism for Treg‐mediated protection from renal IRI.^70^ Tregs suppress innate immune responses through CD73‐mediated dephosphorylation of ATP into adenosine, a molecule that induces anti‐inflammatory effects through binding to A_2a_ receptors (A_2a_R). Compared to the adoptive transfer of wild‐type Tregs, transfer of Tregs from CD73 and A_2a_ receptor (A_2a_R)‐deficient mice into wild‐type mice prior to IRI resulted in reduced Treg function and increased renal injury.^73^ Microarray analysis revealed that activation of Treg A_2a_R significantly enhanced PD‐1 expression, which was required for Treg's effects in IRI, implying that autocrine adenosine signalling assists Tregs in suppressing innate immune responses in IRI via PD‐1.^73^ Both PD‐1 ligands (PD‐L1 and PD‐L2) are instrumental in protecting the kidney from IRI.^74^

Given the protective role of Tregs in renal IRI, a number of pharmacological, biological or non‐Treg cellular therapies that target and/or recruit intrinsic Tregs to the kidney have been employed *in vivo*. Pretreatment of mice with the sphingosine kinase inhibitor, *N*,N‐dimethylsphingosine (DMS), rapidly and transiently recruits CD4^+ ^Foxp3^+^ Tregs and CD4^+ ^Foxp3^−^ cells to the kidney and prevents IRI.^75^ These protective effects were Treg and CTLA4 dependent.^75^ Bone marrow‐derived mesenchymal stem cells (MSCs) also ameliorate renal IRI by increasing Treg proportions in the spleen and ischaemic kidney, effects dependent on both an intact spleen and on Tregs.^76^ Transfer of human‐umbilical cord blood‐derived MSCs has similar effects.^77^ Other interventions potentially mediated by modulating Tregs include a protective role for microRNA 26a (Mir‐26a), which plays functional roles in cell differentiation, growth, apoptosis and metastasis, and modulates Th17/Treg balance,^78^ and a P2X7 receptor antagonist, periodate‐oxidised ATP (oATP).^79^ As in other experimental models, in IRI IL‐2/anti‐IL‐2 mAb complexes administered prior to IRI increased Tregs (in the spleen and kidney), resulting in less renal dysfunction and tubular injury, and when given after IRI, they promoted functional recovery and inhibited renal fibrosis.^80^ As IL‐2 and IL‐33 promote the expansion of murine Tregs *in vivo*, Stremska *et al*. generated an IL‐2 and IL‐33 fusion cytokine that they termed IL‐233, and which they found increased the recruitment of Tregs into the kidney and protected mice from IRI more efficiently than either cytokine alone.^81^ Thus, these studies collectively suggest that strategies aimed at enhancing numbers, recruitment and function of endogenous Tregs demonstrates therapeutic potential in AKI, especially as therapies prior to injury in situations where AKI is likely or probable.

### Cisplatin nephrotoxicity

Cisplatin, an inorganic platinum‐based chemotherapeutic agent, is widely used in the treatment of many solid organ malignancies. However, its use is limited by the significant incidence of (approximately 25–35%) nephrotoxicity.^82^ Cisplatin concentrates in the epithelial cells of the S3 segment of proximal tubules, where it induces both necrotic and apoptotic cell death with an accompanying substantial pro‐inflammatory immune response.^83^ CD4^+ ^Foxp3^+^ Tregs are protective in experimental cisplatin nephrotoxicity, where they migrate to the kidney as early as 6 h after injury.^84^ As in IRI, studies that have either depleted endogenous Tregs or adoptively transferred Tregs (into immunodeficient or immunocompetent mice) have demonstrated a protective role for Tregs in cisplatin nephrotoxicity,^3^, ^84^ Mechanistically, Tregs are likely to have their effects, at least in part, by suppressing macrophage infiltration and innate immune responses. While the detailed mechanisms of Treg recruitment to the kidney in AKI remain unclear, one study used a series of reconstitution and depletion studies in wild type, *Rag1* ^*−/−*^ and Foxp3^DTR^ mice to demonstrate that Toll‐like receptor 9 (TLR9)‐deficient Tregs fail to effectively localise to the kidney following cisplatin administration, resulting in enhanced renal injury and dysfunction.^3^ The impaired Treg recruitment in the absence of TLR9 was due to impaired adhesion molecule expression on Tregs.^3^ However, the details of the underlying mechanism by which Tregs influence the intrarenal innate immune response and promote renoprotection in cisplatin nephrotoxicity remain unclear.

As in other disease models, a variety of interventions have been linked to enhance Treg number or function in cisplatin nephrotoxicity. Interestingly, bee venom injections, with the active component being phospholipase A2 (PLA2), a calcium‐dependent lipolytic enzyme before the administration of cisplatin increase Treg numbers in the spleen and enhance their recruitment during the early phase of cisplatin‐induced nephrotoxicity, reducing renal dysfunction and intrarenal inflammation, accompanied by reduced intrarenal IL‐6 and TNF.^85^, ^86^ The effects of PLA2 were dependent on the presence of Tregs, and mediated through binding to mannose receptor CD206 on dendritic cells, inducing IL‐10 production.^86^ The renoprotective effects of human‐umbilical cord blood‐derived MSCs administered early after cisplatin administration are potentially via Tregs.^87^ As in IRI, the hybrid cytokine IL‐233 protected mice from cisplatin‐induced AKI, but whether this was mediated through increased recruitment of Tregs to the kidney (as in IRI) was not determined.^81^ Given that patients with solid organ tumours are given cisplatin in a known timeframe, IL‐233 as well as PLA2 and MSCs, assuming they do not limit the anticancer effects of cisplatin may have therapeutic potential.

### Sepsis‐induced acute kidney injury

Sepsis, a systemic inflammatory response to infection, is a common cause of AKI.^88^ Inflammation, oxidative stress, microvascular dysfunction and tubular epithelial responses are involved in the pathogenesis of this complex and multifactorial syndrome.^89^ Patients with septic AKI have increased serum soluble CD25 and IL‐10 that is strongly associated with immunosuppression.^90^ Similarly, in a mouse model of caecal ligation and puncture (CLP)‐induced sepsis, septic AKI increased Treg numbers, immune cell apoptosis and IL‐10 levels.^91^ In contrast to IRI and cisplatin nephrotoxicity, depletion of Tregs before CLP with anti‐CD25 antibody was renoprotective and resulted in better survival, highlighting a paradoxical immune effect of Tregs in AKI secondary to sepsis syndrome.

## Regulatory T cells in chronic kidney disease

The development of glomerulosclerosis and progressive interstitial fibrosis and tubular atrophy is a common feature of many types of renal disease. These progressive lesions are a function of persistent inflammatory, metabolic or genetic insults, but when disease is advanced and functional nephron number reduced beyond a critical threshold, progressive fibrosis occurs even in the absence of ongoing insults. Thus, modulation of Tregs may have a place not only in switching off the disease causing insults, but might also have direct and beneficial components on fibrosis within the kidneys themselves.

### Adriamycin nephropathy

Adriamycin nephropathy in rodents is a reproducible model of chronic kidney disease induced by the chemotherapeutic agent adriamycin. This model is characterised by focal segmental, global glomerular sclerosis, podocyte fusion and severe proteinuria that subsequently causes tubulointerstitial fibrosis and inflammation.^92^ Although it remains unclear whether there is a role for antigen‐specific cells in this model, both T and B lymphocytes, as well as macrophages, mediate disease progression. The depletion of CD4^+^ T cells in established adriamycin nephropathy exacerbates glomerular and tubulointerstitial injury, suggesting that a CD4^+^ regulatory subset may inhibit disease progression.^93^ Following on from this, adoptive transfer experiments using Foxp3‐transduced CD4^+^ cells protected mice from adriamycin nephropathy, and anti‐CD25 antibodies exacerbated disease.^94^ Transfer of CD4^+ ^CD25^+^ Tregs into SCID mice with established adriamycin nephropathy also reduced glomerular and interstitial injury associated with a marked decline in intrarenal macrophage numbers, suggesting a direct effect on renal mononuclear phagocytes independent of adaptive immunity.^95^ *In vitro* mechanistic studies suggested that the lymphocyte‐independent protective effect of Tregs was mediated via a TGF‐β‐dependent Treg‐macrophage inhibitory interaction.^95^ The transfer of a subset of activated M2 macrophages generated *ex vivo* IL‐10/TGF‐β was protective in established AN.^96^ Treated mice exhibited reduced renal fibrosis, associated with reduced macrophage infiltration and increased Tregs in the draining lymph nodes, with Treg depletion abolishing these protective effects.^97^ Thus, it is likely that in chronic kidney disease, Tregs mediate protection by direct effects on innate immune cells, particularly macrophages, and on the injured kidneys themselves. However, despite the absence of direct evidence for antigen‐specific events in this model, when Tregs from TcR transgenic mice were transferred into immunocompetent mice, they did not limit adriamycin‐induced renal injury.^98^ Tregs also appear to mediate protection in adriamycin nephropathy via increased CD39 expression, with CD39‐overexpressing mice protected against renal injury, and transfer of CD39Tg Tregs being highly effective in limiting renal damage in adriamycin nephropathy.^99^ Expanding Tregs with IL‐2/anti‐IL‐2 complex *in vivo* also reduced renal dysfunction and inflammation, even after the onset of AN.^100^

### Diabetic nephropathy

Diabetic nephropathy, occurring as a result of the autoimmune disease type 1 diabetes mellitus (T1DM) or the more metabolically defined type 2 diabetes mellitus, is a major complication of these diseases and is the leading cause of end‐stage renal disease worldwide. DN is characterised by glomerular hypertrophy, basement membrane thickening, the accumulation of extracellular matrix components and kidney inflammation that is crucial in promoting the development and progression of DN.^101^ While autoimmune pancreatic β‐cell destruction causes insulin deficiency that leads to T1DM, there is little evidence for autoimmunity in the nephropathy that results from many years of DM. While it is unclear whether patients with DN have altered Treg numbers, Foxp3^+^ Tregs are increased fivefold in the renal interstitium of mice with streptozotocin‐induced T1D compared with nondiabetic wild‐type mice.^102^ However, these results could conceivably be confounded by the fact that streptozotocin is a tubular toxin. In T2DN, Tregs may modulate renal inflammation and disease severity. Patients with T2DN have reduced levels of CD4^+ ^CD25^+ ^Foxp3^+^ Tregs in the periphery, which negatively correlates with the urine albumin:creatinine ratio.^103^, ^104^, ^105^ In db/db mice with T2D, Treg depletion using anti‐CD25 antibodies enhanced insulin resistance, albuminuria and glomerular filtration, whereas the adoptive transfer of CD4^+ ^CD25^+ ^Foxp3^+^ Tregs improved insulin sensitivity and diabetic nephropathy with increased Foxp3 mRNA expression in both the kidney and visceral adipose tissue.^106^ Despite these studies suggesting a possible link between Foxp3^+^ Tregs and disease progression in DN, further investigation is required to understand their precise role for more targeted therapeutic options.

## Regulatory T cells in renal transplantation

Renal transplantation is clearly the optimal therapy for end‐stage kidney disease. While rates of early graft loss falling over the past 15 years, long‐term graft survival and toxicities of immunosuppression remain as major issues. Regulatory T cells in renal transplantation are highly relevant, not only because of their importance in potentially establishing graft tolerance, but also as the timing of renal transplantation allows for isolation, expansion and infusion of a patient's own Tregs. As in autoimmune diseases, a protective role for Tregs is implied by a number of observational studies in human renal transplantation, backed by functional evidence in experimental renal transplantation. In addition, in human renal transplantation a number of clinical trials of Treg therapy are underway (e.g. in the ONE Study, www.onestudy.org). Early reports show the feasibility of expanding nTregs with allogeneic DCs^107^ and support the safety of cell therapy with nTregs.^108^ Table 2 lists some of the differences between the use of human and mouse Tregs.

**Table 2:** Comparison of some of the features of human and mouse Tregs

Feature	Human	Mouse
Surface markers	CD4^+^ CD25^+^ CD127^lo/−^ CD4^+^ CD25^+^ CD127^lo/−^ CD45RA^+^ CD4^+^ CD39^+^ CD4^+^ CD25^high^ CD6^lo^	CD4^+^ CD25^high^
Use of Foxp3	Functional studies cannot be performed with Foxp3 as a marker	Foxp3 reporter mice
Isolation	Autologous fresh peripheral blood and FACS	Splenocytes and/or lymph nodes and FACS (higher purity than MACS)
Expansion	*Ex vivo* polyclonal expansion of CD4^+^ CD25^hi^ CD127^lo^ with anti‐CD3 and anti‐CD28 paramagnetic beads with IL‐2	*Ex vivo* polyclonal expansion with CD4^+ ^CD25^hi^ from naïve mice with anti‐CD3 and anti‐CD28 microbeads with high dose IL‐2

FACS, fluorescence‐activated cell sorting; MACS, magnetic‐activated cell sorting.

In something of a contrast to disease in native kidneys, the relative frequency of biopsy of transplanted kidneys (including via ‘protocol biopsies’) has allowed a more detailed assessment of intrarenal Tregs. While Tregs are present in and around tubules in grafts, intragraft Tregs in aggregates are present in some human renal transplants. These aggregates correlate both with long‐term graft survival and relative donor hyporesponsiveness.^109^ Not all studies assessing Treg numbers or FOXP3 mRNA have demonstrated these associations, suggesting that Treg numbers increase in inflammatory states and that Treg:Teff ratios might be more predictive. Alternately, FOXP3 can be expressed by effector cells in humans raising the question of whether the FOXP3^+^ cells demonstrated in renal allografts are in fact functionally Tregs. While these cells have been reported to exhibit demethylation of the Treg‐specific demethylated region (TSDR) at the FOXP3 locus,^110^ kidney transplant patients do exhibit a variable degree of TSDR demethylation in FOXP3^+^ cells,^111^ implying that ongoing allogeneic stimulation may influence the phenotype and stability of Tregs. Several cytokines produced by Tregs, including IL‐10, TGF‐β and IFN‐γ, have been associated with good outcomes in renal transplantation.^112^, ^113^, ^114^, ^115^, ^116^, ^117^

Experimentally, tolerance in murine kidney allografts is Treg dependent and can be established by Treg transfer.^2^, ^118^ Tregs in grafts from tolerant mice with the ability to transfer tolerance expressed TGF‐β, IL‐10, IFN‐γ, Blimp‐1 and Cxcr3.^2^ Immature renal dendritic cells can induce IL‐10 producing Tregs *in vitro*,^119^ emphasising the need to minimise innate inflammation, such as prolonged warm ischaemic times at transplantation, as IRI can activate intrarenal mononuclear phagocytes to enhance their antigen‐presenting functions.^120^ Renal tubular cells may also be important in influencing suppressive Treg phenotypes and behaviours in transplantation.^121^

While some details of Treg biology in renal transplantation are unclear and Treg stability (at least in some Treg subsets) may be a challenge, infusion of Tregs has real therapeutic potential in renal transplantation. Successful Treg therapy would at least allow significant reduction in drug therapy and may establish tolerance or operational tolerance to the graft. Clearly, unlike autoimmune renal disease, the timing of transplantation allows the infusion of Tregs prior to allogenic stimulation at the time of surgery. After nTreg isolation, both nonantigen‐specific and allogeneic stimulation and expansion protocols are being employed, including the use of belatacept (CTLA 4‐Fc) *ex vivo*.^107^, ^122^ These protocols have been generating cells that maintain their phenotype including demethylation of the FOXP3 TSDR.^107^ Furthermore, Tr1 cells can also be generated from patients with end‐stage kidney disease who are on dialysis.^123^

A further dimension in the role of Tregs in transplantation is the effect of current and new immunosuppressive agents on Tregs and their interactions with Tregs. Current regimens, while effective, have not evolved with consideration of their effects on Tregs. If Treg therapy finds a place in renal transplantation, it would ideally be combined with therapies that do not themselves negatively affect Treg function *in vivo*, for example, mTOR inhibitors, low‐dose IL‐2 or histone deacetylase inhibitors.

## Subsets of suppressive T cells

The focus of this review has been on the role of CD4^+ ^Foxp3^+^ Tregs in renal disease. However, several other T‐cell subsets with suppressive activity have been identified. These include CD4^+ ^Foxp3^−^ T cells that secrete IL‐10, TGF‐β and IL‐35 and are termed Tr1, Th3 and Tr35, respectively.^124^, ^125^, ^126^ In addition, other suppressive cells not belonging to the Th lineage include CD3^+ ^CD4^− ^CD8^−^ double‐negative T cells, Qa‐1‐restricted CD8 Tregs and CD28^lo^CD8^+^ Tregs.^127^, ^128^, ^129^ While these additional regulatory T‐cell subsets have not been as well studied as the conventional CD4^+ ^Foxp3^+^ Tregs that are discussed in this review, there is evidence for their protective involvement in renal disease.^27^, ^123^, ^130^, ^131^, ^132^

## Conclusion

While the detailed phenotype of Tregs within the kidney remains understudied, Tregs, both systemic and local, play a major role in limiting many forms of renal injury. These include conditions that would intuitively be Treg responsive, such as the growing number of autoimmune forms of glomerulonephritis and renal transplantations, but also conditions with less obvious links to innate immunity, for example acute kidney injury. Treg therapy is being trialled in renal transplantation and has potential in other renal diseases, and interventions that promote the number or function of Tregs also are possible future treatments for a variety of diseases that affect the kidney.
